# Strengthening Anti-Glioblastoma Effect by Multi-Branched Dendrimers Design of a Scorpion Venom Tetrapeptide

**DOI:** 10.3390/molecules27030806

**Published:** 2022-01-26

**Authors:** Wassim Moslah, Dorra Aissaoui-Zid, Soioulata Aboudou, Zaineb Abdelkafi-Koubaa, Marie Potier-Cartereau, Aude Lemettre, Ines ELBini-Dhouib, Naziha Marrakchi, Didier Gigmes, Christophe Vandier, José Luis, Kamel Mabrouk, Najet Srairi-Abid

**Affiliations:** 1 Laboratoire des Biomolécules, Venins et Applications Théranostiques (LBVAT), LR20IPT01, Institut Pasteur de Tunis, Université de Tunis El Manar, Tunis 1002, Tunisia; aissaoui.dorra@yahoo.com (D.A.-Z.); abdelkafi_zaineb@yahoo.fr (Z.A.-K.); ines.bini@pasteur.tn (I.E.-D.); naziha.marrakchi@pasteur.tn (N.M.); 2 Institut de Neurophysiopathologie (INP), UMR 7051-CNRS, Faculté de Médecine, Aix-Marseille Université, 27 bd Jean Moulin, 13385 Marseille, France; jose.luis@univ-amu.fr; 3 Institut de Chimie Radicalaire (ICR), Aix-Marseille Université, CNRS, ICR UMR 7273, 13397 Marseille, France; soioulata@hotmail.fr (S.A.); didier.gigmes@univ-amu.fr (D.G.); kamel.mabrouk@univ-amu.fr (K.M.); 4 N2C UMR 1069, INSERM, Faculté des Sciences et Techniques, Université de Tours, 37032 Tours, France; marie.potier-cartereau@univ-tours.fr (M.P.-C.); aude.lemettre@univ-tours.fr (A.L.); christophe.vandier@univ-tours.fr (C.V.)

**Keywords:** glioblastoma, chemical synthesis, multi-branched peptide, dendrimers, AaTs-1, anti-proliferative effect

## Abstract

Glioblastoma is the most aggressive and invasive form of central nervous system tumors due to the complexity of the intracellular mechanisms and molecular alterations involved in its progression. Unfortunately, current therapies are unable to stop its neoplastic development. In this context, we previously identified and characterized AaTs-1, a tetrapeptide (IWKS) from *Androctonus autralis* scorpion venom, which displayed an anti-proliferative effect against U87 cells with an IC_50_ value of 0.57 mM. This peptide affects the MAPK pathway, enhancing the expression of p53 and altering the cytosolic calcium concentration balance, likely via FPRL-1 receptor modulation. In this work, we designed and synthesized new dendrimers multi-branched molecules based on the sequence of AaTs-1 and showed that the di-branched (AaTs-1-2B), tetra-branched (AaTs-1-4B) and octo-branched (AaTs-1-8B) dendrimers displayed 10- to 25-fold higher effects on the proliferation of U87 cells than AaTs-1. We also found that the effects of the newly designed molecules are mediated by the enhancement of the ERK1/2 and AKT phosphorylated forms and by the increase in p53 expression. Unlike AaTs-1, AaTs-1-8B and especially AaTs-1-4B affected the migration of the U87 cells. Thus, the multi-branched peptide synthesis strategy allowed us to make molecules more active than the linear peptide against the proliferation of U87 glioblastoma cells.

## 1. Introduction

Glioblastoma multiform (GBM) is a type IV brain tumor, representing the most aggressive form of primary cerebral cancers [1]. It affects astrocytes cells, which have considerable roles in the cerebral tissue [2]. Due to their substantial invasive and migratory capacities, GBM cells can infiltrate the healthy parts of the cerebral cortex [3]. They mainly attack other brain sites, seriously altering their microenvironment, although extracranial metastasis remains extremely rare [4].

Given the large glioblastoma cells genomic and phenotypic diversity, diagnoses are often unfavorable. Often, therapies proposed for GBM patients consist of surgery associated with radiotherapy and chemotherapy. The chemotherapy strategy includes molecules such as temozolomide (TMZ) and carmustine [4,5]. However, these treatments remain underperforming due to their non-specificity and the hyperinvasive aspect of glioblastoma cells in the cerebral cortex. During the development of glioblastoma, a variety of mutations and complex hallmarks are acquired [6] and induce patient resistance to the conventional treatments [4]. The most common mutations are those that affect genes coding for VEGF (vascular endothelial growth factor) and its receptor VEGFR, PDGF (platelet-derived growth factor), the tumor suppressor genes NF1, P53, PTEN, and RB *retinoblastoma* proteins [1]. Thus, complex mechanisms contribute to the development of GBM, involving multiple factors and cellular elements such as the angiogenic factors, metalloproteinases, integrins, ionic channels [4], as well as calcium receptors [7,8]. Calcium acts as a second messenger in many molecular mechanisms, and its intracellular rate directly influences glioblastoma cell proliferation and migration [9,10]. Thus, targeting these key cell elements could destabilize tumor development.

In this context, several studies are being conducted to find new drugs against glioblastoma. For instance, biomolecules derived from scorpion venom such as Chlorotoxin (CTX), from the scorpion *Leiurus quinquestriatus,* and its analogs have been shown to inhibit proliferation, invasion and/or angiogenesis of glioblastoma cells. CTX is a chloride channel modulator shown to interact specifically and with high affinity with glioblastoma cells [11,12]. Moreover, a novel CTX-like peptide, namely AaCtx, was purified from the venom of the scorpion *Androctonus australis*. It displayed 70% similarity of sequence with CTX in terms of amino acid sequence. Invasion and migration of human glioma cells can be both inhibited by both native and synthetic AaCtx; however, the inhibition activity of AaCtx was lower than that of CTX [13].

Recently a new tetrapeptide named AaTs-1 from the *Androctonus australis* scorpion venom was identified and characterized by our team. It shows a specific effect on U87 glioblastoma cell proliferation with an IC_50_ of 0.57 mM, by acting on calcium flux, likely by modulating the FPRL-1 receptor [14]. Considering its small size and the interesting physicochemical and pharmacokinetic properties of this peptide, we sought, in this work, to improve its activity by designing and synthesizing new molecules, based on its structure, by using the multiple antigen peptide (MAP) technique. This method was initially used with the aim to generate immunogenic peptides in order to sensitize the immune system against antigens [15]. Lately, this technique went beyond this strand and was introduced into several other fields such as an anti-viral molecules [16,17], intracellular transport [18] and platelet aggregation peptides [19].

## 2. Results

### 2.1. Synthesis of AaTs-1 and Dendritic Analogs

**Figure 1:**
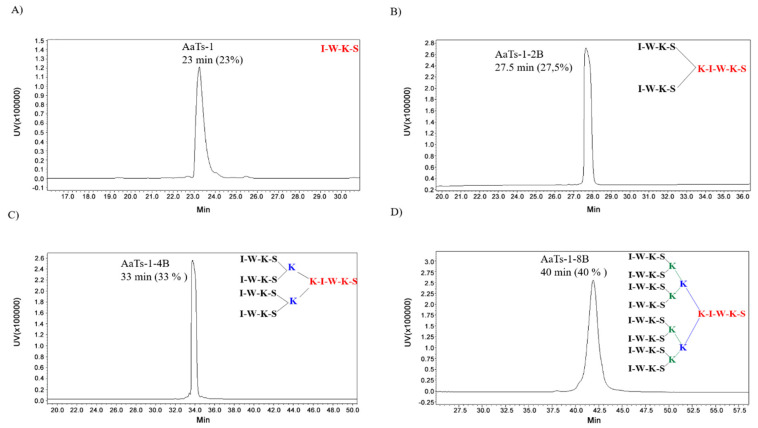
HPLC analytical profiles of AaTs-1 and multi-branched purified peptides. AaTs-1 and MAPs were analyzed after purification on a C18 analytical column and detected at 214 nm. The peptides displayed different retention times due to the structural modifications induced on the sequence of AaTs1. (**A**): AaTs-1 was eluted at 23 min. (**B**): AaTs-1-2B at 27.5 min. (**C**): AaTs-1-4B at 33.5 min. (**D**): AaTs-1-8B at 41 min. The difference between the 3 dendrimers is the number of branches (insert figures).

In order to design and synthesize new multi-branched peptides based on the AaTs-1 peptide structure, we used the MAP strategy. The di-([IWKS]2-K-βA)., tetra-([IWKS]4-[K]2-K-βA) and octo-([IWKS]8-[K]4-[K]2-K-βA) branched peptides were assembled using the Fmoc/tbu strategy on an HMP resin. The synthesis of these analogs is based on a system of branched lysines linked by different sequences of the linear peptide in their N-terminal extremity, thus giving these dendrimers a three-dimensional conformation different from the starting peptide [15]. Branched dendrimers structures contain three components, as shown in Figure 1 (inserts). The first one consists of the initial sequence, followed by a polylysine core and then several copies of the IWKS sequence 2, 4 and 8 motifs for the di-branched, tetra-branched and octo-branched respectively (Figure 1, inserts).

All peptides were purified (Supplementary Materials; Figure S1) and characterized by electrospray mass spectroscopy: AaTs-1 MW = 533 Da, expected MW = 532.64; AaTs-1-2B MW = 1690 Da (Table S1, supplementary materials), expected MW = 1688.98; AaTs-1-4B MW = 2976 Da (Table S2), expected MW = 2974.75; and AaTs-1-8B MW = 5547 Da (Table S3), expected MW = 5545.30. The purity of the molecules were checked by analytical HPLC. They displayed different retention times due to the structural modifications induced on the sequences of AaTs1. AaTs-1 was eluted at 23 min; AaTs-1-2B at 27.5 min; AaTs-1-4B at 33 min; AaTs-1-8B at 40 min (Figure 1).

### 2.2. In Vivo Effect of AaTs-1 and Dendrimers

All mice injected by AaTs-1 or dendritic analogs remained alive even 24 h after the injection. Furthermore, no signs of toxicity or changing behavior were recorded for the two doses of 1 and 4 µg for any of the molecules. This result demonstrated that the molecules are not toxic *in vivo.* AaTs-1 and the dendrimers are at least 8000 times less toxic, compared to AaHII (LD_50_ = 0.5 ng), the most toxic molecule of *Androctonus autralis* scorpion venom [20].

### 2.3. Effect of AaTs-1 and the Dendrimers on U87 Cells Viability

**Figure 2:**
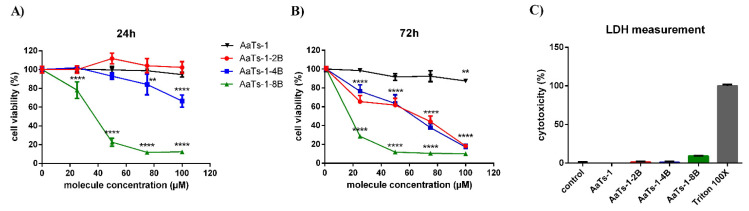
Effect of AaTs-1 and the dendrimers on U87 cells viability. U87 cells were incubated with different concentrations of AaTs-1, AaTs-1-8B, AaTs-1-4B or AaTs-1-2B for 24 h (**A**) or 72 h (**B**). Cells viability was measured using MTT method. (**C**) Cells were treated by AaTs-1, dendrimers or triton X-100 for 24 h. LDH in supernatant was measured at 460 nm according to the kit manufacturer protocol. The percentage of cytotoxicity is estimated by dividing the OD obtained with each molecule by the OD of positive control (Triton X-100)**.** The results are representative of three independent experiments. ** and **** denote *p* < 0.01 and 0.0001, respectively, versus negative control.

The effect of the synthesized peptides on the viability of U87 cells was first evaluated by MTT assay. Results showed that both AaTs-1 and AaTs-1-2B had no effect on U87 cells after 24 h treatment, while AaTs-1-4B inhibited slightly their viability by 33.7% at 100 µM. However, the most important inhibition was obtained with the octo-branched molecule (AaTs-1-8B), with an IC50 of 31.94 µM (Figure 2A).

The assessment of the molecules effect on the proliferation rate of U87 cells (viability after 72 h) showed that the multi-branched molecules inhibited the U87 cells proliferation with IC_50_ values of 22.7, 48.2, and 51.5 µM for AaTs-1-8B, AaTs-1-4B, and AaTs-1-2B, respectively (Figure 2B), while AaTs-1 had almost no effect (Figure 2B).

Particularly, we noted that AaTs-1-2B and AaTs-1-4B displayed roughly the same effect on the proliferation assay mirrored by an almost equivalent IC_50_. As a whole, our results showed that the modifications we performed on AaTs-1 structure increased by at least 10 times the effect on U87cell proliferation. AaTs-1at 100 µM showed only 12.7% inhibition, while the multi-branched molecules inhibited U87 cell proliferation by more than 80% at the same concentration (Figure 2B).

The LDH release assay, quantifying the release from cell of LDH enzyme when the membrane is perforated, was performed to determine the mechanism of death undergone by U87 cells after their treatment for 24 h with AaTs-1 (at 100 µM), AaTs-1-2B (50 µM), AaTs-1-4B (50 µM), and AaTs-1-8B (30 µM). The molecule concentrations were chosen for their closeness to their respective IC50 on U87 cells viability determined by MTT assay. Our results showed that the peptides AaTs-1, AaTs-1-2B and AaTs-1-4B had no effect on LDH release at the concentration used, whereas AaTs-1-8B induced a slight release (9.1%) (Figure 2C). At this percentage, AaTs-1-8B is considered not toxic in U87 cells [21].

### 2.4. Videomicroscopy Migration Assay

**Figure 3:**
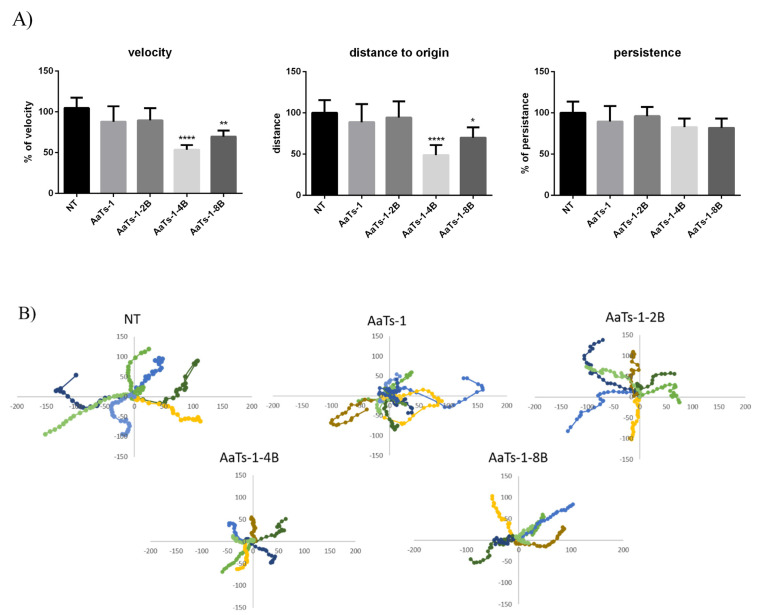
Effect of AaTs-1 and dendrimer analogs on migration of U87 cells using a time-lapse microscopy. U87 cells were seeded at 10^4^ cells/well on fibronectin pre-coated 24-well plate and treated with non-toxic concentrations of the synthetic molecules. Pictures of five fields per well were taken at 5 min intervals during 4 h. (**A**) Cell velocity, directional persistence and distance to origin were calculated from time-lapse videomicroscopy. (**B**) Representative migration paths of 10 cells are reported by using position parameters, each color corresponds to the trajectory of one cell. Each result is representative of three independent experiments. *, ** and **** denote *p* < 0.05, 0.01 and 0.0001, respectively, versus negative control.

Cell migration is a complex and important phenomenon that allows aggressive cells to invade other sites and initiate metastasis. Therefore, the effect of branched analogues on U87 cell migration was tested at the selected concentrations by performing a two-dimensional migration assay using timelapse microscopy [22,23]. Several parameters were calculated (Figure 3A), and a representative graph of the trajectories (where the origin of each cell track has been fixed at the coordinates x = 0, y = 0) followed by 10 cells is presented in Figure 3B. Results showed that AaTs-1 and AaTs-1-2B did not significantly affect migration of fibronectin, while AaTs-1-4B significantly reduced the motility of U87 cells more than AaTs-1-8B. The trajectory of cells treated by AaTs-1-4B and AaTs-1-8B was modified and the distance to origin was affected. The analysis of trajectory performed on each cell indicated that the velocity of cells treated by AaTs-1-4B and AaTs-1-8B was reduced by 46% and 25%, respectively, compared to untreated cells.

### 2.5. Western Blot Analysis

**Figure 4:**
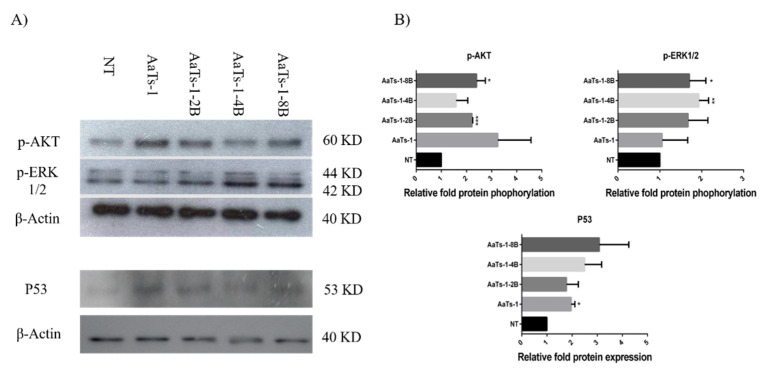
Western blot analysis. (**A**) Cells were treated or not (NT) with AaTs-1 (100 µM), AaTs-1-2B (50 µM), AaTs-1-4B (50 µM) or AaTs-1-8B (10 µM) for 30 min to evaluate the MAPK phosphorylation, AKT and ERK1/2, and for 24 h to evaluate the expression of P53. Cells were then lysed by Laemmli buffer and analyzed by Western blot. (**B**) Relative protein phosphorylation/expression level, normalized to control, was presented on a graph bar generated by Image-J software. Statistical analysis and histograms were created with Prism 8 software. Student’s *t* test was used with a *p* value < 0.05 to be considered significantly different to control condition (* *p* < 0.05; ** *p* < 0.01 and *** *p* < 0.001).

To identify the pathways implicated on the observed effects of AaTs-1 and dendrimers, we performed Western blot analysis of the main proteins involved in major pathways of oncogenesis. Our data showed that AaTs-1 and dendrimers exerted a remarkable elevation in the expression of P53 protein. Furthermore, all the molecules upregulated the phosphorylation form of AKT and ERK after 30 min treatment, compared to control (Figure 4). We observed that the dendrimers induced a more consistent increase in ERK1/2 phosphorylation than AaTs-1, indicating that the modifications made on the structure of AaTs-1 molecule, by increasing the number of IWKS motifs, act directly on its mode of action.

### 2.6. Effect of AaTs-1 and Dendrimers on Cytosolic Calcium Concentration

**Figure 5:**
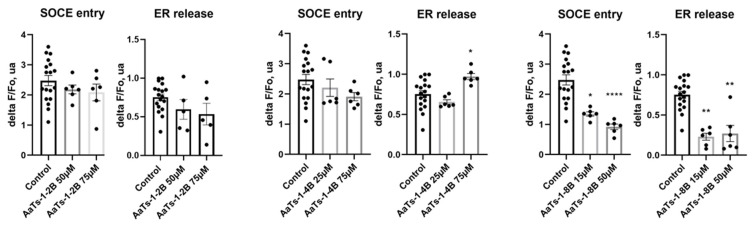
Effects of acute application of molecules on the release of ER and amplitude of SOCE in U87 cells. Cells were loaded with fura-2-AM. Amplitude of fluorescence ratio variation, in comparison to control condition, was measured. Individual values and the mean value +/− SEM of n observations (*N* = 3) are presented in the different histograms. Statistical analysis and histograms were created with Prism 8 software. Kruskal–Wallis test and Dunn’s test were used with a *p* value < 0.05 to be considered significantly different from control condition (* *p* < 0.05; ** *p* < 0.01; and **** *p* < 0.0001).

AaTs-1 was previously shown to decrease the intracellular calcium concentration, probably by upregulating FPRL-1 receptor expression. We thus checked the effect of the dendrimers on the cytosolic calcium concentrations and the endoplasmic reticulum (ER) calcium release to the cytoplasm by using the fluorescent probe Fura-2-AM. Our results revealed that AaTs-1-2B did not show any effect on cytosolic calcium concentration control by store-operated calcium entry (SOCE) and calcium release from ER, whereas AaTs-1-4B increased the ER calcium release without significantly affecting the SOCE, while AaTs-1-8B exerted a significant inhibition of both ER calcium release and SOCE, even at a low concentration of 15 µM (Figure 5).

## 3. Discussion

Despite scientific advances in recent decades, glioblastomas remain aggressive and incurable with classical therapies [24]. Thus, new strategies to develop specific and efficient drugs are needed. In this context, the multiple antigen peptide system (MAP) is one of the successful strategies, introduced in 1988, by J.P. Tam [14], to synthesize a new class of potential pharmaceutic molecules called dendrimers. This process is based on the use of a matrix of lysines, allowing for the grafting of multiple motifs (peptide sequences) on the α and ε amino groups of lysines [24].

Several features have been observed in the mode of action of polylysines dendrimers, including their ability to increase the sensitivity of branched molecules and to strengthen their interactions with their targets [24]. Synthetic dendrimers have a dominant positive overall charge due to the extremities of the repeated units in the branches, allowing them to interact with the cell membrane (or even with DNA), and pass the intracellular compartment [25]. Thus, many efficient dendrimers have been produced, such as gene delivery vehicles, drug carriers, vaccines or anti-viral and anti-microbial molecules [26]. This important aspect prompted us to look for the first multi-branched peptides targeting glioblastoma cells. We aimed to improve the activity of the previously reported scorpion tetrapeptide AaTs-1 (IWKS), which showed antitumor effect on U87 cells [14].

We first checked the ability of dendrimers to decrease the viability of U87 cells and their potential cytotoxic effect, using MTT and LDH tests. MTT results showed that AaTs-1 and AaTs-1-2B, which are the peptides with the fewest IWKS motifs, had no effect on the viability of U87 glioblastoma cells after 24 h treatment. On the contrary, AaTs-1-4B slightly affected the viability, reaching 33.7% of inhibition at 100 µM, while AaTs-1-8B appeared to be the most active molecule, with an IC_50_ of 31.9 µM. Besides, dendritic molecules significantly decreased the viability of U87 cells after 72 h incubation, without being cytotoxic, as assessed by LDH assay. We found that the efficiency of the molecule increased with the number of motifs. Our results are in accordance with those of precedent studies in which chemotherapeutic agents, such as Paclitaxel and Cisplatin, conjugated in stable dendrimers, display higher activities [27]. However, we noted that the effects of di-branched and tetra-branched analogs relative to octo-branched analogs are modest compared to AaTs-1. Thus, there remains the question of what the ideal number of IKWS motif is to have optimal activity. In this context, Martinho et al. [28] reviewed many research papers reporting computational studies of dendrimers for their use as therapeutic agents or in drug delivery. The reported studies showed how computational simulation techniques and models provide a basis to improve our ability to better predict and understand the biological activities and interactions of dendrimers. These include the radial distribution function (RDFs), solvent accessible surface area (SASA) and solvent excluded volume (SEV), radius of gyration (Rg), shape descriptors, counting number of hydrogen pairs, and the mechanistic interactions as well as the thermodynamic parameters associated with them. Together, these features can provide a profile of a given dendrimer [28]. Evangelista-Lara et al. (2005) reported that SASA and SEV examine the molecular surface with a spherical solvent probe to roll around the van der Waals spheres of the macromolecule. This modeling strategy can provide information related to the internal cavities and can be useful to characterize different types of dendrimers and compare which one has the highest potential [29]. However, in our case, it seems that the dendrimers did not interact with the same receptor and may have different and complex mode of action, involving different receptors than those targeted by Aats-1, which could explain why the activity is not proportional to the number of the dendrimer’s branches. This could also explain the new activities acquired by some dendrimers. We found that AaTs-1-4B and AaTs-1-8B significantly inhibited U87 cell migration at 50 and 10 µM, respectively, by decreasing the velocity as well as the distance covered by the glioblastoma cells. It thus appears that, compared to AaTs-1, the multi-branched dendrimers were much more active in U87 cells migration.

To understand the molecular mechanisms of our multi-branched molecules, we studied the expression of P53 and the phosphorylation of ERK1/2 and AKT, known as key effectors of main oncogenic pathways [30]. We observed an activation of P53 protein expression, which correlated with the inhibition of the proliferation of U87 cells obtained with the tested molecules. AaTs-1-8B, the most active of the U87 cell proliferation, induced the highest expression level of P53 protein.

Unexpectedly, we found that all molecules, and especially AaTs-1-8B, induced the increase in the phosphorylated forms of AKT and ERK. The results observed with AaTs-1 peptide are in opposition with those found by Aissaoui-Zid et al. [14]. This variation is found to be due to the difference in the cells treatment time (data not shown), as U87 cells were treated for 30 min in this study, instead of 72 h. Many other studies showed that the phosphorylation levels depend on the cell treatment time [8,31].

Conversely, the increase in the phosphorylated forms of AKT and ERK is generally associated with the development of tumor cells, since their activation is related to mutations affecting upstream oncoproteins [32,33]. Nevertheless, recent studies have shown that the overactivation of AKT and ERK1/2 can generate many responses depending on the pathways implicated. Nogueira et al. have demonstrated that activation of AKT is associated with downregulation of FOXO protein inducing a premature senescence and ROS mediated apoptosis [34]. Conversely, overactivation of ERK1/2 was shown to induce anti-proliferative effects by arresting the cell cycle in G0-G1 phase [35] and causing cell mortality by apoptosis, autophagy or senescence, depending on the mechanisms evolved [36]. Furthermore, in a recent study, Zhang et al. have related an ERK1/2 hyperphosphorylation with anti-proliferative and even an anti-migratory effects [37].

In our case, we found that the synthetic molecules did not induce ROS-mediated apoptosis (Figure S3). Furthermore, Aissaoui-Zid et al. showed that AaTs-1 inhibits U87 cells proliferation without stopping cell cycle process [14]. Thus, these synthetic molecules seem to target a new pathway, which will be interesting to identify.

It was reported that calcium ions play a primordial role in glioblastoma cell motility [38,39]. It has been demonstrated that the expression of modulators of calcium flux on glioblastoma cells, such as FPRL-1 (formyl peptide receptor-like 1), GluRs (Glutamyl-tRNA synthetase) and P2 × 7R (an ATP-gated nonselective cation channel) along with voltage-gated calcium channel, activate the signaling pathways of MAP Kinases, as well as AKT and PI3K, hence triggering survival, growth and mobility of GMB cells [4,40,41].

We thus tested our molecules on cytosolic calcium concentration controlled by SOCE and ER and found that the tetra- and octo-branched dendrimers displayed different activities. AaTs-1-4B increased calcium release by ER without affecting significantly the SOCE, while AaTs-1-8B is the only dendrimer that, at the same time, decreased both SOCE and calcium release from the ER about 50-fold higher than AaTs-1 [14]. Therefore, when compared to the study of Aissaoui-Zid et al., it is obvious that the mechanism of action of AaTs-1 is different from that of AaTs-1-2B, which is also different from those of AaTs-1-4B and AaTs-1-8B.

In conclusion, we showed in this study that the new designed multi-branched dendrimers, based on the sequence of the tetrapeptide AaTs-1, not only increased the effect on the U87 cells proliferation, but also allowed for a new anti-migratory effect.

All the synthetic molecules induced the enhancement of AKT and ERK1/2 phosphorylation and P53 expression. Some of them modulated cytosolic calcium concentration, suggesting the activation of calcium-dependent cellular pathways. Curiously, this effect is generally, but not always, correlated with the number of the IWKS motif copies in the dendrimers. Therefore, it appears that the molecules have different mechanisms of action and may act on different receptors involved in U87 cell proliferation and/or migration. The effect of AaTs-1-8B might correspond to the additive effect of both AaTs-1 and AaTs-1-4B.

## 4. Materials and Methods

### 4.1. Materials

Chemical reagents and amino acids were purchased from Sigma Chemical Company (St. Louis, MO, USA). Medium culture EMEM was from Lonza (Colmar, France) and FBS from Gibco (Cergy-Pontoise, France). The U87 cell line was purchased from the American Type Culture Collection (ATCC, Manassas, VA, USA). Primary and secondary blotting antibodies were purchased from Cell Signaling Technology society.

### 4.2. Animals

Male adult C57BL/6 mice (20 ± 2 g) were purchased from Pasteur Institute of Tunis, housed in clean polypropylene cages and maintained at room temperature of 25 °C with humidity range of 40–70%. The animals were fed with a standard pellet diet and clean drinking water. All procedures were carried out in accordance with the guidelines for care and use of laboratory animals and were approved by bio-medical ethics committee (N° 2015/14/E/FST).

After one-week acclimatization, animals were randomly divided into nine groups based on their body weight.

### 4.3. Chemical Synthesis of AaTs-1 and MAPs

All peptide syntheses performed in this work are based on FMOC/tBu chemistry using an automatic synthesizer (Model 433A, Applied Biosystems) on an HMP (4-hydroxymethylphenol) CHEMMATRIX resin (0.65 mmol/g), as solid support, in DCM. The two ([IWKS]2-K-βA), four ([IWKS]-[K]2-K-βA) and eight IWKS motifs ([IWKS]8-[K]4-[K]2-K-βA) were anchored on an uncharged lysine-based core matrix as described previously [42]. Briefly, after coupling the first amino acid to the resin in the presence of DCC, HBTU/HOBt, and DIPEA, several cycles of deprotection and coupling were repeated to assemble amino acids successively. The deprotection of each amino acid amine function was carried out with an NMP/piperidine solution (4v/1v). The final cleavage of the synthesized peptide and the deprotections of side chains of the amino acids and the amino part of the last amino acid linked was carried out in a solution of TFA, H_2_O and Tri-isopropylsilane as scavenger (v: 95%, v: 2.5%, v: 2.5%). The solution containing the synthesized peptide was filtrated to remove the resin, and then the peptide was precipitated with cold ether. Following the same protocol, the linear AaTs-1 peptide was first synthesized and then the dendritic, octo-branched (AaTs-1-8B), tetra-branched (AaTs-1-4B) and di-branched (AaTs-1-2B) analogs, based on the MAP (multiple antigen peptide) strategy [15].

The synthetic AaTs-1 was purified by semi-preparative RT-HPLC (NUCLEODUR 100-5 C18 ec 250 × 10 mm, MACHERY NAGEL) using a linear gradient from 0% to 60% (60 min) of solvent B (90% can, 10% H_2_O, 0.1% TFA) in A (0.1% TFA in water), at a flow rate of 4 mL/min. The multi-branched peptides were purified on a preparative HPLC C18 column (NUCLEODUR 100-5 C18 ec 250 × 21 mm, MACHERY NAGEL), using a linear gradient from 0% to 80% (80 min) of solvent B at a flow rate of 6 mL/min. The homogeneity and identity of the synthesized molecules were assessed by analytical LC-MS, using a HPLC (LC-2010AHT, Shimadzu) linked to electrospray mass spectrometer.

### 4.4. In Vivo Toxicity Assay

AaTs-1 and dendritic analogs were tested for in vivo toxicity on male C57/BL6 mice, by intracerebroventricular (ICV) route. Freshly prepared solutions containing each synthetic molecule at 1 or 4 µg in 0.1% (*w*/*v*) BSA in water were tested. Three mice were used per dose for each molecule and three others as control, injected with only 0.1% BSA in water. ICV administration was performed under anesthesia conditions, according to the method described elsewhere [43].

Mice were followed for 24 h to determinate the mortality rate and/or any behavior modification, especially tremors, lethargy and anxiety. Experiments on mice were carried out in accordance with the European Community Council Directive (2010/63/EU) for experimental animal care, and all procedures met with the approval of the Institutional Research Board of the Pasteur Institute of Tunis.

### 4.5. Cell Culture

U87 cells were routinely cultured in EMEM (Eagle’s Minimum Essential Medium) (Gibco™, Cergy-Pontoise, France; Sigma, St. Louis, MO, USA) supplemented with 10% fetal bovine serum (FBS), 1% L-glutamine and 100 IU/mL penicillin/streptomycin. Cells were maintained at 37 °C in a humid atmosphere of 5% CO_2_.

### 4.6. Cell viability Assessment

MTT assay

U87 cells were seeded at 10^4^ or 5 × 10^3^ cells per well overnight and then treated by different concentrations of the synthetic molecules for 24 or 72 h. Then, cells were incubated for 3 h with MTT (0.5 mg/mL). Afterward, DMSO was added to solubilize formazan crystals and the OD was measured at 600 nm to quantify the percentage of living cells. IC_50_ were calculated using Prism Graphpad software.

LDH release assay

Cellular membrane integrity was monitored by the permeability assay based on the release of lactate dehydrogenase (LDH) into the media. LDH release from U87 cells was determined by using the LDH Cytotoxicity Detection Kit-PLUS test (Roche Applied Science, Mannheim, Germany) according to the manufacturer protocol. Briefly, cells were seeded at 3 × 10^5^ in 6-wells plate and treated by the synthesized molecules, at their respective IC_50,_ or Triton X-100, as positive control, for 24 h. The concentration of LDH was evaluated by the measuring the absorbance at 460 nm, after addition of a mixture of substrate and dye solution provided by the kit.

### 4.7. Videomicroscopy Migration Assay

A 24-well plate was coated with fibronectin matrix at 10 µg/mL in PBS. Cells were seeded (10^4^ cells/well) for 2 h at 37 °C until they adhered and then treated with different concentrations of the synthetic molecules and incubated at 37 °C with 5% CO_2_. Five fields in each well were photographed every 5 min for 4 h using a Coolsnap HQ camera (Photometrics, Tucson, AZ, USA) operated by NIS-elements AR 2.30 software (Nikon, New York, NY, USA). Results were analyzed by Metamorph^®^ image analysis software (Roper Scientific, Evry, France). Several parameters were calculated for each cell, including total migration distance from origin, velocity, and directional persistence of cell migration. Total migration distance represents the sum of distances between each measurement. The distance to origin was determined as the net translocation between the initial and the final position over a period of 4 h. Velocity was calculated as the total migration distance divided by 4 h. Directional persistence of cell migration is considered as the ratio of the distance to origin to the total distance migration. For each parameter, results are expressed as the mean ± SD from at least 30 individual cells [23,44].

### 4.8. Cytosolic Ca^2+^ Measurements

To evaluate the effect of the synthesized molecules on the calcium flux, we measured cytosolic calcium concentration using the fluorescent ratiometric dye Fura-2-AM from Thermo Fischer Scientific (Illkirch, France). U87 cells were seeded at 5 × 10^4^ in a 96-well plate overnight and acutely treated with AaTs-1 peptide or its dendritic analogues and then loaded with Fura-2-AM (1 µM) for 45 min at 37 °C. A Flexstation 3 microplate reader (Molecular Devives, San Jose, CA, USA) was used to measure fluorescence (excitation 340 and 380 nm; emission 510 nm). In order to measure cytosolic calcium release from the endoplasmic reticulum (RE), cells were stimulated without extracellular calcium, with tapsigargin (4 µM) at 100 s of recording, and 2 µM CaCl_2_ was added at 500 s to measure store-operated calcium entry (SOCE). After normalization to control conditions, SOCE amplitude and cytosolic release by ER release were measured by calculating the ΔF/F0 ratio. Analyses were performed using SoftMax Pro Software (5.4.6 version, Molecular Devices, San Jose, CA, USA).

### 4.9. Western Blot Assay

Cells were treated by the synthetic molecules and lysed using Laemmli buffer and then denatured at 96 °C for 5 min. Cell lysates were centrifuged at 12,000× *g* RPM for 5 min at 4 °C. The concentration of proteins recovered in the supernatant was determined using BCA protein assay. Afterward, 30 µg of proteins of each lysate were separated by SDS-electrophoresis using 10% polyacrylamide gel and transferred to a polyvinylidene difluoride (PVDF) membrane. After saturation with 5% of non-fat dried milk, membranes were incubated with first antibody at 1/1000 in milk solution overnight at 4 °C, washed with PBS 0.1% Tween and exposed to the second antibody (1 µL/10 mL) for 1 h at room temperature. Finally, the membranes were washed and revealed using chemi-luminescence detection system (ECL, X-OMAT EX II Developer and Replenisher, KODAK, Rochester, NY, USA).

### 4.10. Statistical Analysis

Data were analyzed using *t* test and ANOVA one-way with Kruskal–Wallis test to evaluate significance between three tests. Data were presented as mean ± SD. *p* value < 0.05 was considered significant. MTT IC_50_ was calculated by GraphPad Prism6 considering shapes of curves and SD.

## Data Availability

AaTs-1 sequence was obtained from publication under name “AaTs-1: A Tetrapeptide from *Androctonus australis* Scorpion Venom, Inhibiting U87 Glioblastoma Cells Proliferation by p53 and FPRL-1 Up-Regulations” (*Molecules* 2021, *26*, 7610. https://doi.org/10.3390/molecules26247610 SM, accessed on 20 November 2021), and the sequence data will appear in the UniProt Knowledgebase under the accession number C0HLZ5.

## Supplementary Materials

The following supporting information can be downloaded online. Figure S1: Preparative HPLC profiles of multi-branched dendrimers. (A) AaTs-1-2B (B) AaTs-1-4B, (C) AaTs-1-8B. The blue curve corresponds to the absorbance at 214 nm while the red curve represents the absorbance at 280 nm. The peaks of interest are surrounded by black rectangles. Figure S2: Molecular mass spectra of multi-branched dendrimers. (A) AaTs-1-2B: masses detected 845, 564 and 423. (B) AaTs-1-4B: mass detected 596. (C) AaTs-1-8B: masses detected: 1111, 925, 793 and 694. Figure S3: measurement of reactive oxygen species (ROS) production. (A) Representative histogram showing flow cytometry data of measurement of ROS production with CMH2DCFDA staining after 2 h of treatment with AaTs-1 (100 µM), multi-branched analogs AaTs-1-2B (50 µM), AaTs-1-4B (50 µM) and AaTs-1-8B (30 µM) and LPS (1 µM) as control. (B) Quantitative histogram of median fluorescence intensity proportional to ROS production. Table S1: Theoretical molar mass of AaTs-1-2B. Table S2: Theoretical molar mass of AaTs-1-4B. Table S3: Theoretical molar mass of AaTs-1-8B.

## Sample Availability

Samples of the compounds AaTs-1, AaTs-1-2B, AaTs-1-4B and AaTs-1-8B are available from the authors.
