# Supplementary material for: Strengthening Anti-Glioblastoma Effect by Multi-Branched Dendrimers Design of a Scorpion Venom Tetrapeptide
Source: Molecules. 2022 Jan 26;27(3):806. doi: 10.3390/molecules27030806 (PMC8838298; doi:10.3390/molecules27030806)
Supplement: Supplementary file 1 [file molecules-27-00806-s001.zip › molecules-1508412-supplementary.pdf]

Supplementary materials

Strengthening anti-glioblastoma effect by multi-branched dendrimers design of a scorpion venom tetrapeptide

Wassim Moslah [1,2], Dorra Aissaoui-Zid [1], Soioulata Aboudou [3], Zaineb Abdelkafi-Koubaa [1], Marie Potier-Cartereau [4], Aude Lemettre [4], Ines ELBini-Dhouib [1], Naziha Marrakchi [1], Didier Gigmes [3], Christophe Vandier [4], José Luis [2], Kamel Mabrouk [3], Najet Srairi-Abid [1].

[1]LBVAT, Laboratoire des Biomolécules, Venins et Applications Théranostiques, LR20IPT01, Institut Pasteur de Tunis, Université de Tunis El Manar 1002 Tunis, Tunisie.

[2]INP, Institut de Neurophysiopathologie; UMR 7051-CNRS & Aix-Marseille Université, Faculté de Médecine, 27 bd Jean Moulin, 13285 Marseille cedex 05, France.

[3]ICR, Institut de Chimie Radicalaire; Aix Marseille Univ, CNRS, ICR UMR 7273, F-13397 Marseille, France.

[4]N2C UMR 1069, University of Tours, INSERM, 37032 Tours, France.

Figure S1

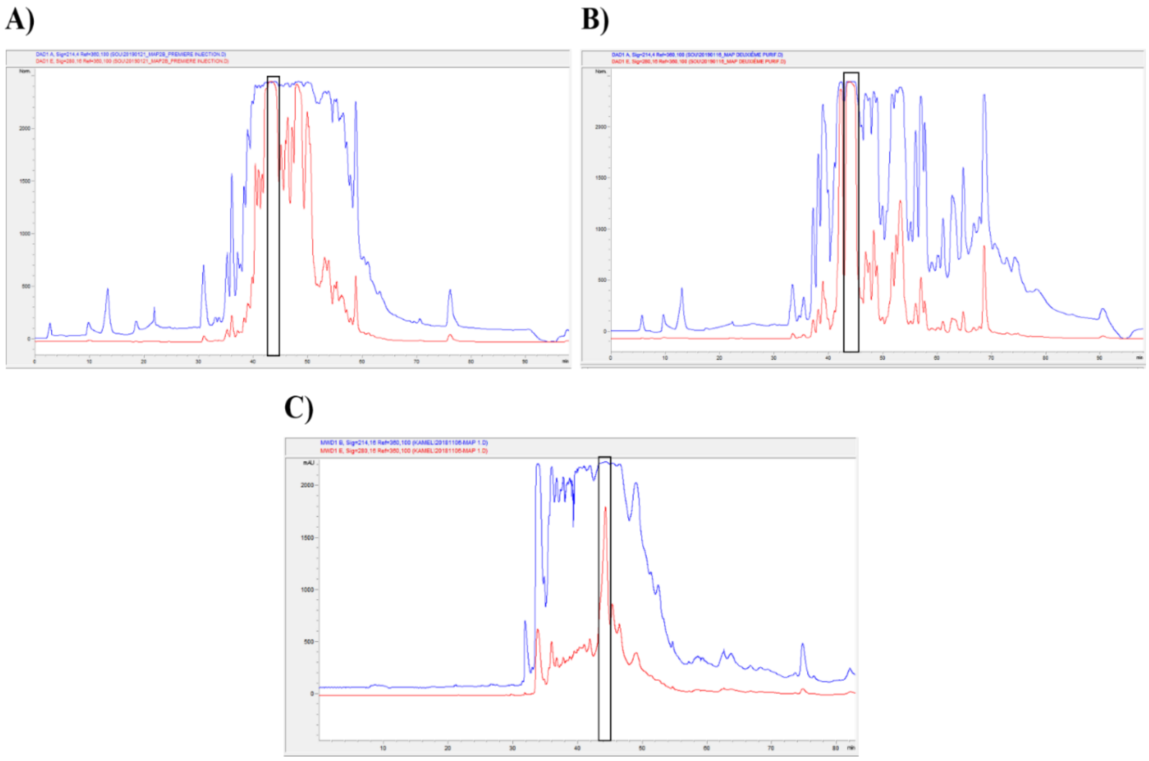

Figure S1. Preparative HPLC profiles of multi-branched dendrimers. (A) AaTs-1-2B (B) AaTs-1-4B, (C) AaTs-1-8B. The blue curve corresponds to the absorbance at 214 nm while the red curve represents the absorbance at 280 nm. The peaks of interest are surrounded by black rectangles.

Figure S2

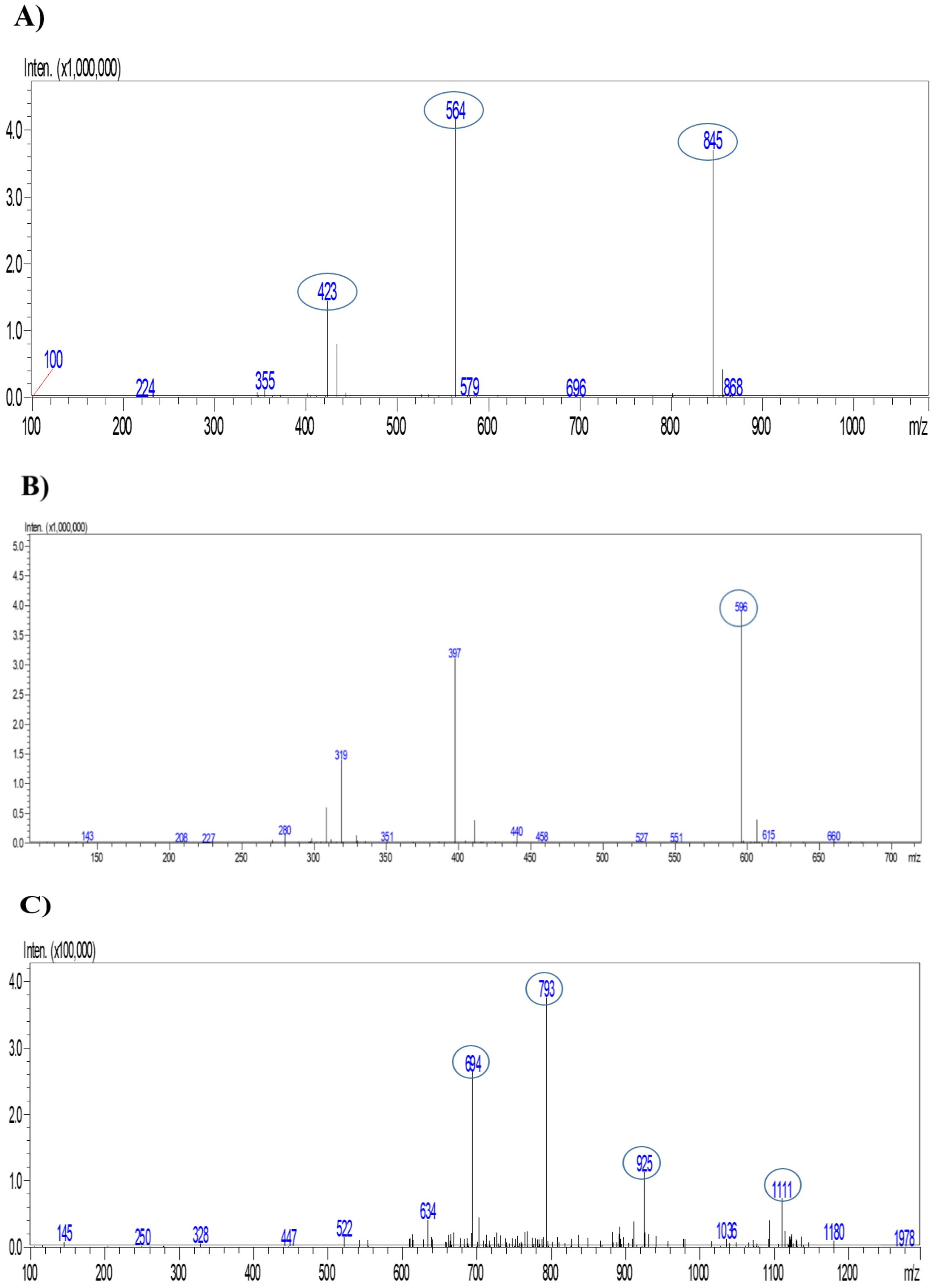

Figure S2. Molecular mass spectra of multi-branched dendrimers. (A) AaTs-1-2B: masses detected 845, 564 and 423. (B) AaTs-1-4B: mass detected 596. (C) AaTs-1-8B: masses detected: 1111, 925, 793 and 694.

Table S1. Theoretical molar mass of AaTs-1-2B.

Charges	Masses
Z=1	1690
Z=2	845.5
Z=3	564
Z=4	423.25
Z=5	338.8

Table S2. Theoretical molar mass of AaTs-1-4B.

Charges	Masses
Z=1	2977
Z=2	1489
Z=3	993
Z=4	745
Z=5	596.2
Z=6	496.66
Z=7	426.14
Z=8	373

Table 3. Theoretical molar mass of AaTs-1-8B.

Charges	Masses
Z=1	5548
Z=2	2774.5
Z=3	1850
Z=4	1387.75
Z=5	1110.4
Z=6	925.5
Z=7	793.22
Z=8	694.37
Z=9	620.33
Z=10	555.7

Figure S3

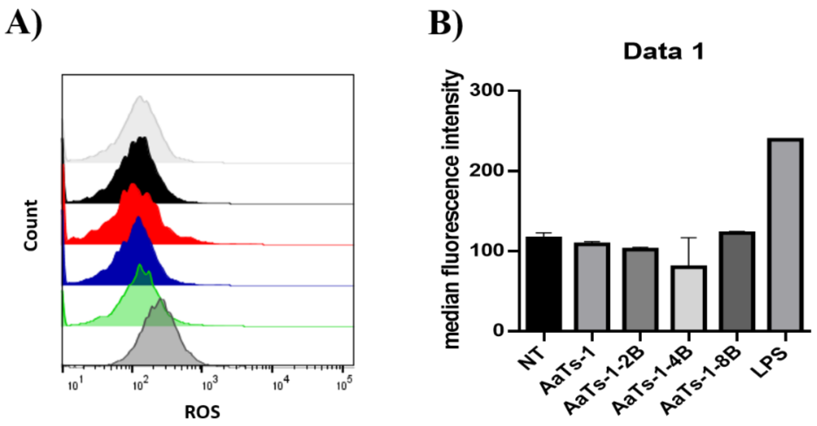

Figure S3. Measurement of reactive oxygen species (ROS) production. (A) Representative histogram showing flow cytometry data of measurement of ROS production with CMH2DCFDA staining after 2 h of treatment with AaTs-1 (100 µM), multi-branched analogs AaTs-1-2B (50 µM), AaTs-1-4B (50 µM) and AaTs-1-8B (30 µM) and LPS (1 µM) as control. (B) Quantitative histogram of median fluorescence intensity proportional to ROS production.
